# Mottling score and skin temperature in septic shock: Relation and impact on prognosis in ICU

**DOI:** 10.1371/journal.pone.0202329

**Published:** 2018-08-16

**Authors:** Arnaud Ferraris, Camille Bouisse, Nicolas Mottard, Fabrice Thiollière, Sophie Anselin, Vincent Piriou, Bernard Allaouchiche

**Affiliations:** 1 Service d’Anesthésie-Réanimation, Centre Hospitalier Lyon Sud, Hospices Civils de Lyon, Lyon, France; 2 Université Lyon 1 Claude Bernard, Lyon, France; Hospital Universitari Bellvitge, SPAIN

## Abstract

**Introduction:**

Mottling score, defined by 5 areas over the knee is developed to evaluate tissue perfusion at bedside. Because of the subjective aspect of the score, we aimed to compare mottling score and skin temperature in septic shock with infrared thermography in ICU and the correlation to survival.

**Methods:**

We conducted a prospective and observational study in a teaching hospital in France during 8 months in ICU. All patients with sepsis requiring vasoactive drugs were included. We recorded epidemiologic data, hemodynamic parameters, mottling score and skin temperature with a thermic camera of the 5 mottling areas around the knee (temperatures recorded with FLIR™ software) at bedside. Measures were performed at ICU admission (H0) and six hours after initial resuscitation (H6).

**Results:**

46 patients were included. Median age was 69 (60–78), SOFA score 11 (8–12) mean SAPS II was 57±20 and 28-day mortality rate was 30%. Patients with mottling (score≥1), had a skin temperature of the knee significantly lower (30.7 vs 33,2°C p = 0.01 at H6) than patients without mottling (score = 0). Skin temperatures of the knee in mottling groups 1 to 5 were similar at H0 and H6. Neither mottling score nor skin temperature of the knee were associated with prognostic regarding day-28 mortality.

**Conclusions:**

Skin temperature measured with infrared thermography technology around the knee is lower when mottling sign is present and sign microcirculation alterations. This method, compared to standard mottling score is objective and allows data collections. However, this method failed to predict mortality in ICU patients.

## Introduction

Microcirculation is a transport system responsible for the delivery of oxygen from blood flow to tissues of the body [1]. Circulatory shock reduces perfusion and oxygenation to tissues, resulting in hypoxia and finally organ failure [2]. Microcirculatory disorders take an important place in physiopathology of septic shock and have been studied in many studies [3,4]. Microcirculatory monitoring is feasible directly at patient bedside (mottling score, diuresis, skin temperature gradient) or with most sophisticate methods (sublingual videomicroscopy with sidestream dark field (SDF) which evaluate microvascular perfusion [5], and indirectly with near-infrared spectroscopy (NIRS) which mostly evaluates muscle perfusion and detects global oxygenation variations in tissue perfusion [6]). None of these techniques has showed superiority on the other. There is no gold standard to compare any of these methods, which makes microcirculatory evaluation very difficult. We decided to focus on skin perfusion of the knee by mottling score, basing our choice on previous studies [7].

**Fig 1:**
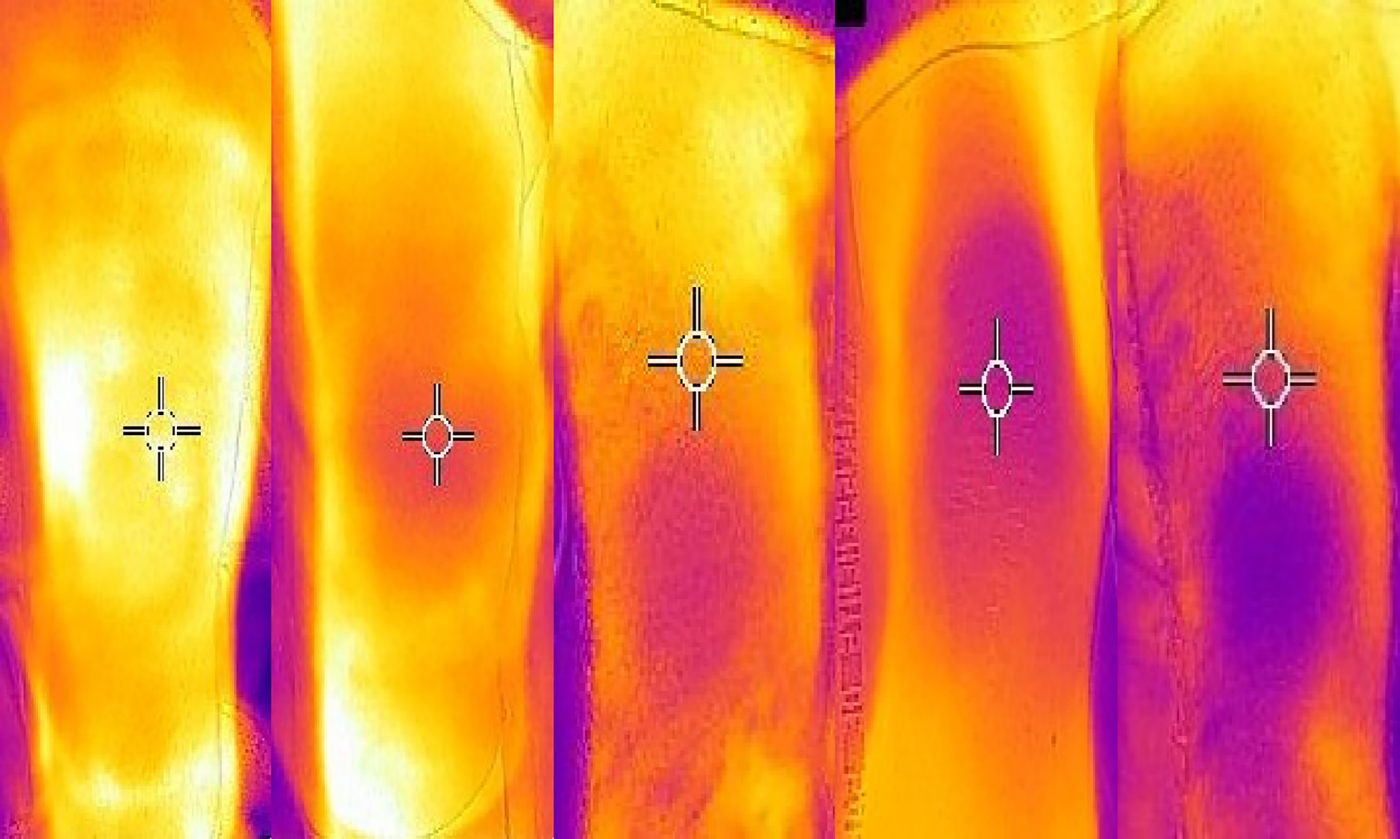
**Examples of thermography, from mottling score 0 to 4 (from left to right)**.

Skin mottling is a violaceous coloration secondary to microcirculation alterations and heterogeneous blood-flow reduction in small vessels, an example is presented in Fig 1 with a schema of the five mottling zones. In a recent observational study, mottling incidence were 29% in a non-selected cohort of 791 patients admitted in ICU and increase to 49% in patients with sepsis. Admission severity score as SAPS II were greater in mottled patients compare to those free of mottling (mean SAPS II 46 (34–59) versus 32 (21–45) respectively) [8]. It is a clinical sign of circulatory shock [2], easy to describe by physician over the knee and identified as an unfavorable outcome in critically ill patients [9]. However, mottling score is a subjective clinical sign and unrealizable in many patients.

**Fig 2:**
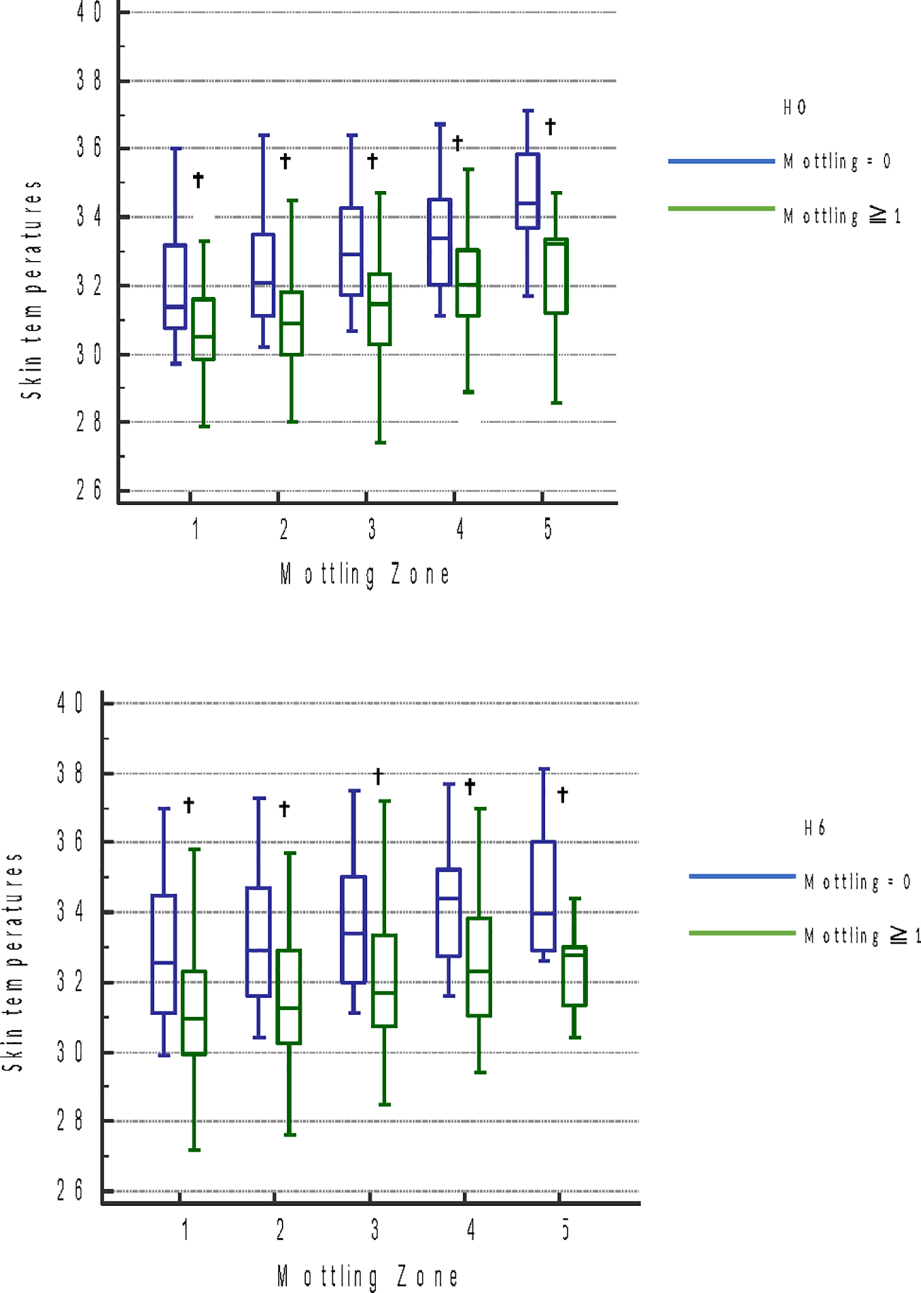
Skin temperatures according to presence or absence of mottling in the different mottling zones at HO and H6.

Infrared thermography is a non-invasive technology with an infrared camera that record infrared radiations emitted by the body and deduce a temperature. The infrared camera creates images based on differences in surface temperature by detecting infrared radiation that emanates from objects and translate temperatures in a color gradient in a photography (Fig 2). Infrared thermography offers a useful and non-invasive approach to the diagnosis of many disorders such as circulatory abnormalities, but few studies describe this technique [10].

We assumed skin knee temperatures are correlated to mottling score. To our knowledge, no study investigated objective skin temperatures and thermographic measurements in septic shock with comparison to mottling score. We conducted a prospective observational study including all patients admitted in ICU for septic shock. We compared mottling score with skin temperature on the knee using infrared thermography. We also evaluated correlation between these values and survival.

## Materials and methods

We conducted a prospective observational study in an intensive care unit (ICU) of the teaching hospital of Lyon (France) during a 8-month period from January to august 2016. We included consecutive patients, ≥18 years old, admitted for septic shock defined as a suspected sepsis and the systolic arterial pressure less than 90 mmHg or the mean arterial pressure less than 65 mmHg despite of fluid resuscitation, and requiring vasoactive drugs at admission in ICU. Exclusion criteria were black skin, pregnancy, obliterating arteriopathy of the lower limbs, cutaneous infection of the lower limbs and patient or family refusal.

As the design of the study was purely observational, waive written informed consent was authorized (local Ethics Committee). Verbal information was given to all patients. Institutional approval was obtained from the local Ethics Committee (Comité de Protection des Personnes sud est IV Lyon, France; Ref L15-2013, approved on December 15 of 2015). As the design of the study was purely observational, waive written informed consent was authorized. Verbal information was given to all patients or family according to their level of consciousness.

### Endpoints

Primary endpoint is a positive correlation between mottling score and skin temperatures in thermography (at the place corresponding to mottling zones) during a septic shock. Secondary endpoints are relationship between skin temperatures, ICU mortality, 28-day mortality, severity score on admission and discharge and vasopressor requirements.

### Management of patients

Patient management with sepsis shock was guided by local protocol derived from international guidelines [11]. Management was not modified by the measurement of skin temperature of the knee through infrared camera. For patients admitted for septic shock, intravenous volume expansion with crystalloids and initiation of norepinephrine were provided at admission to achieve following end-points: mean arterial pressure more than 65 mmHg (or 70 mmHg in patients with chronic arterial hypertension) and urine output > 0.5mL/kg/h. Minimal equipment were provided with invasive arterial monitoring, central venous catheter and ventilation support when needed. Advanced hemodynamic monitoring was provided with transthoracic echocardiography (Vivid S6, General Electric Healthcare®) and/or with transpulmonary thermodilution (PiCCO, Pulsion medical system®). Central venous saturation monitoring was not standard of care excepted for cardiogenic failure. Sedation and analgesia, if required, were performed with midazolam and sufentanil respectively. Hydrocortisone (200mg per day) could be used during septic shock at the discretion of the clinician.

### Protocol and data collection

General characteristics of the patients were recorded: age, sex, severity of illness evaluated by the Sequential Organ Failure Assessment (SOFA) and Simplified Acute Physiologic Score II (SAPS II) scores calculated on admission [12,13]. We collected the following data at study inclusion (H0) and 6 hours following inclusion (H6): general hemodynamic parameters (mean arterial pressure, heart rate, cardiac index), arterial lactate levels, mechanical ventilation requirement, vasopressor doses, mottling score (from 0 to 5), central temperature and room temperature.

Thermography was performed at admission (H0) and 6 hours following inclusion (H6) with a thermic camera FLIR-E® at bedside, centered on one knee (identical at H0 and H6), vertically 50 cm from the bed. Skin temperatures (°C) recorded from the camera software (FLIR® Systems, Inc) were: central knee temperature and global temperatures from the 5 mottling zones respectively. Prospectively we recorded one point (central knee point) and five surfaces matching to the five mottling zones. Surfaces were identically delineated with the camera software modeled from mottling zones and mean temperatures were recorded. We also investigated skin temperature differences in patients with mottling (score ≥ 1) and patients without mottling (score = 0) at H0 and H6.

Central temperature probably impacts on skin temperature, in consequence we investigated this point with five temperature gradients from central to mottling zones (T central–T zone), and mottling zone temperatures evolution between H6 to H0 (T zone H6 –T zone H0).

Finally, ICU and 28-day mortality rate, length of stay, levels of vasopressor and ventilation free-days, SOFA score on discharge were recorded.

### Statistical analysis

Data are presented as mean ± standard deviation (SD) or median (25^th^-75^th^) for non-normally distributed variables (Shapiro-Wilk test) or number (%), as appropriate. Quantitative data between groups were compared using ANOVA with post-hoc tests. The one-way analysis of variance (ANOVA) was used to determine whether there were any statistically significant differences between the means (temperature) of 5 independent (unrelated) groups (mottling score). We considered a p value <0.05 as statistically significant. Statistical analysis was performed with the JMP 13.1 software (SAS institute).

## Results

We included 46 patients prospectively during a 8-month period in ICU for septic shock. General characteristics of the patients are reported in Table 1. All patients received vasopressor therapy but only 3 patients were weaned in the H6 time point after initial resuscitation. The ICU-mortality rate was n = 14 (30%) at day-28. Among patients, only 25 (54%) had mottling (score ≥ 1) at H0 and 30 (65%) at H6 of the management.

**Table 1:** General characteristics.

Patients (n)	46	
Age, years	69 (59–78)	
Male gender	21 (45%)	
Body Mass Index (kg/m2)	24 (21–28)	
SOFA at admission	11 (8–12)	
SAPS II at admission	57 (± 20)	
**Hemodynamic parameters**	**H0**	**H6**
Mean arterial pressure(mmHg) Cardiac output (mL/min) Volume expansion (mL)	67 (± 13) 4.9 (4.5–5.4) 1000 (750–2250)	73 (± 12) 5.1 (4.2–5.7) 1000 (100–2000)
Norepinephrine doses(μg/kg/min)	0.37 (0,13–0,8)	0.42 (0.15–0.9)
Lactate level (mg/L)	2.4 (1.2–4.7)	2.2 (1.4–4)
Mechanical ventilation	31 (67%)	
**Outcomes**		
ICU length-of-stay, days 28-day mortality	12 (5–24) 14 (30%)	

Values are expressed as absolute values (n), %, mean (± SD) or median (25^th^-75^th^).

We compared mottling scores (H0 and H6) to skin temperatures in the five mottling zones, data are shown in S1 Table. Skin temperatures of each different mottling zone of the knee were similar at H0 and H6.

In the group of patients with mottling (score ≥ 1) skin temperature was significantly lower than patients without mottling (score = 0) at H0 and H6 (Fig 2). Temperature gradients from central temperature to knee temperature, and evolution of these gradients between H0 and H6 were similar for patients with mottling (data not presented).

**Fig 3:**
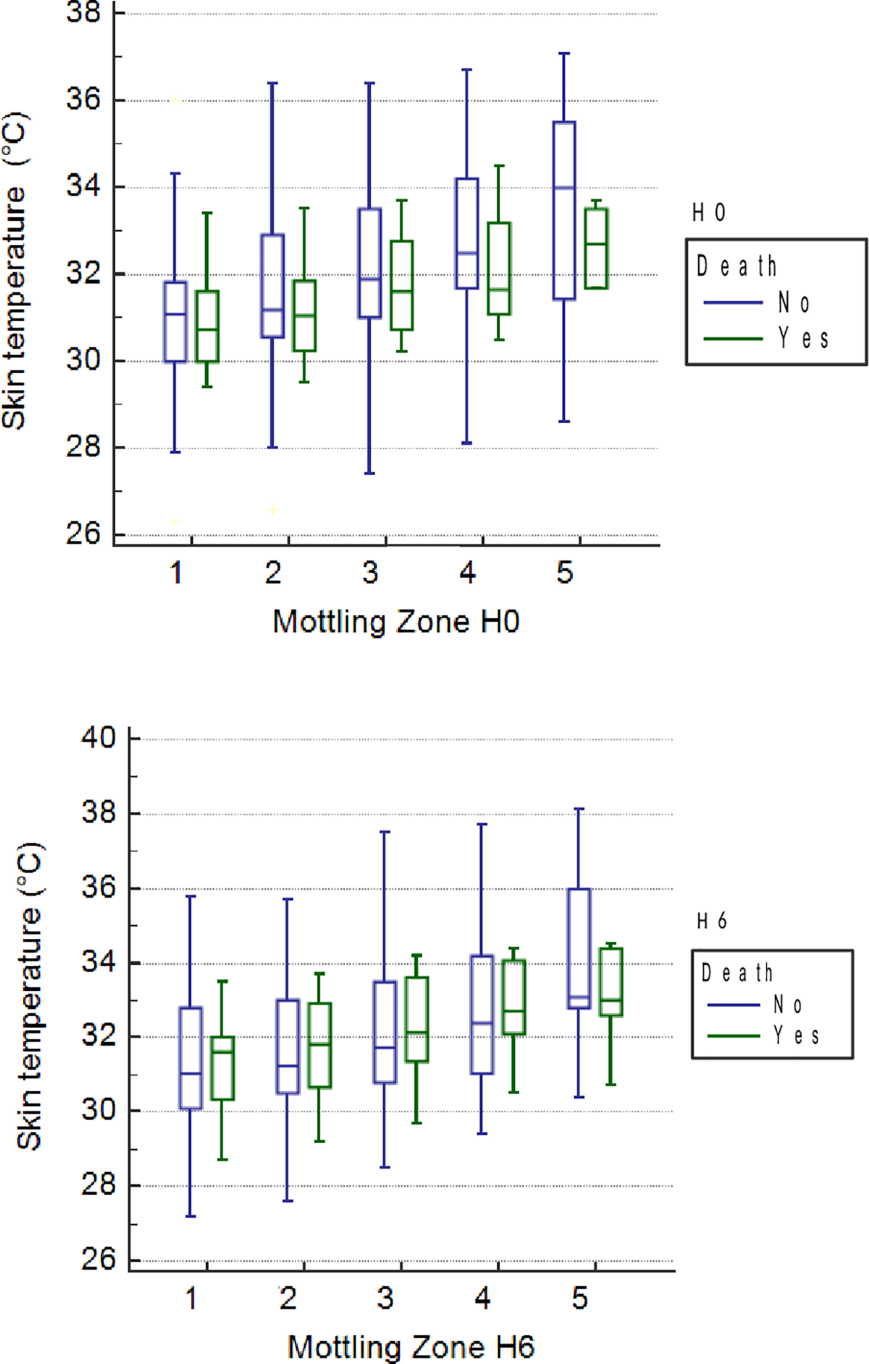
Skin temperature according to 28-day mortality in the different mottling zones at H0 and H6.

Of the patient with mottling (score ≥ 1) at H0 or H6, day-28 mortality was not different from patients without mottling (score = 0) (Table 2). No correlation between mottling score and mortality could be established. Skin temperatures of the knee were similar in survivors and non-survivors (Fig 3). Among risk factors of ICU mortality, mean SOFA score at admission was not different between survivors and dead (10.7±0.9 vs 10.3±1.4; p = 0.79), such as SAPS II (54.7±3.2 vs 64.3±5; p = 0.13), lactate level at H0 (2.6±0.6 vs 4.7±0.9; p = 0.13), lactate level at H6 (2.9±0.7 vs 5.5±1.2; p = 0.07) and in vasopressor-free days (13.3±2.8 vs 3.6±4.2; p = 0.06). We found a difference in the hemodynamic SOFA score (0±0.2 vs 3±0.2; p<0.0001), SOFA score at ICU discharge (1±0.5 vs 11±0.7; p<0.0001) and in ventilator-free days (6.8±1.1 vs 1.1±1.6; p = 0.005) between survivors and dead respectively.

**Table 2:** Presence or absence of mottling correlated to day-28-mortality at H0 and H6.

H0	Survivors	Dead
Mottling ≥ 1	20 (45%)	10 (23%)
Mottling = 0	10 (23%)	4 (9%)
		p = 0.75
**H6**		
Mottling ≥ 1	16 (47%)	9 (26%)
Mottling = 0	6 (18%)	3 (9%)
		p = 0.89

Data are expressed as n (%).

## Discussion

We conducted a prospective observational study to establish a link between skin mottling and skin temperatures in septic shock patients using a thermographic technology. Patients with mottling (score≥1) have skin temperatures significantly lower than patients without mottling (score = 0). This result is reported in the different mottling zones of the knee independently from mottling score. This result is not impacted by central temperature, temperature gradients from central to skin are not different in mottled patients. Furthermore, mottling score and skin temperature were not associated in this study with ICU and day-28 mortality. This study shows the feasibility of thermography to assess microcirculation, as the mottling score at bedside, without subjectivity of the clinician.

Microcirculatory alterations in septic shock have effects on skin temperature. In 80s, Schringer and Baraff confirmed the central part of skin temperature as microcirculation parameter to determine capillary refill time normal thresholds [14]. Since many studies confirmed microvascular alterations in circulatory shock, and most of them focused in sepsis shock [15,16]. Nevertheless, microvascular blood flow alterations are present in various critical situations: cardiogenic shock [17], severe trauma [18]. Thereby, we included in this study only patients with sepsis shock to create a homogeneous cohort of sepsis, expression of microcirculatory abnormalities involved in its complex pathogenesis.

Numerous microcirculation explorations are described in literature, some are clinical assessments easy to realize but often not objective measurements: capillary refill time [19], toe-to-room temperature gradients [20], mottling score [9]. Other techniques as microvideoscopy, orthogonal polarization spectral or sidestream dark field imaging evaluate directly microvascular networks [5]. However, these techniques are in development, sometimes expensive and not routine practice at bedside. As there is no gold standard to compare with, we decided to focus to a clinical, simple, rapid and reproducible test feasible at bedside. Mottling score, well-described and studied by Ait-Oufella answer these characteristics and is correlated to mortality and ICU-outcomes in septic shock [9]. In this monocentric study, authors included 60 patients in septic shock and analyzed the relationship between 14-day mortality and mottling score. They reported the mottling score as a strong predictor of ICU-mortality (p<0.0001), as well as its variation between admission H0 and H6. That is why we compared objective measurements with this clinical sign. Since mottling evaluation is not feasible or not reliable in back skins, we excluded from this study black skins to compare mottling scores to thermography. In this study, thermography technique was employed: a thermic camera records infrared rays and converts it into an electronic signal, which is then processed to produce a thermal image on which you can perform temperature calculations by a software (FLIR® Systems, Inc). This type of thermic camera provides imaging details with an excellent resolution (320x240 pixels), sensitivity (<0.10°C) and accuracy < 2% (manufacturer specifications). We didn’t calculate the coefficient variation, only one measure was performed. However, according scientific literature this coefficient is very limited (1 to 2%) [21]. Currently, few studies reported this technique in medical literature except vascular surgery [10]. The hypothesis was microcirculatory disorders, like mottling, are detectable in thermography because of skin temperatures variations.

We measured skin temperature in the knee, and defined a strict study protocol to standardized all acquisitions. No data in the medical literature helped to build a protocol. Consequently, manufactory recommendations were used: one meter distance from the leg, vertically, emissivity of 0.98. Room’s and patient’s temperatures were measured before thermography to exclude bias from environment or fever and to assure comparability in groups. The side was identical at H0 and H6 at clinician discretion, we assumed no difference were significant between right and left side, excluding severe arteriopathy or trauma.

Study time points (H0 and H6) were empirically fixed according to data from mottling score explorations [9,22]. Indeed, these time points were the most correlated to mortality. H0 time is the worst microcirculation situation, and at H6 point microcirculation situation is stabilized for most of patients after initial resuscitation.

The main objective of the study was to establish a relationship between mottling and skin temperature. The first result is there is no correlation, as the mottling did not influence the skin temperature. Nevertheless, when there is mottling the temperature is lower. Secondly, this study prove thermography feasibility with an objective value without operative dependent measure. Third, in our study ICU mortality wasn’t impacted by mottling score.

Subjective evaluation of peripheral circulation is a valid option and remains first line exploration. The systematic use of most sophisticated material, such as thermography is probably not desirable, but this technique can help in second intention in selected situations. Thermography can help in complex situations in suspected septic patients to admission decision in ICU. Because thermography failed to predict ICU outcomes in this study with a small sample size, this method remains in medical and scientific research field.

This study is in contradiction with previous studies [9,22]: no correlation was found between ICU outcomes and skin temperatures neither mottling score. These studies, observational with a limited cohort included only septic shock. This present study is the first negative study concerning mottling and balance published studies. We reported the absence of correlation firstly between skin temperature and 28-day mortality, and secondly in traditional ICU outcomes as SOFA and SAPS scores. This second point corroborates the lack of power hypothesis in our study. Furthermore, ICU scores such as hemodynamic SOFA, SOFA at ICU discharge or lactate level (p = 0.07 at H6) are different or almost between dead and survivors. By contrast correlation between skin temperature and mortality in one hand, or mottling score and mortality on the other hand is inexistent in our study. We can explain this lack of results with a limited patient cohort, the study was not designed to show a difference in mortality but to compare mottling score and thermography technology. Other limitations are notable though to explain lack of results: it is a monocentric study, included patients are limited, without previous calculation of the number of subjects required, regarding of the lack of data in this topic. No randomization nor blind was respected. Furthermore, reference was mottling score, a most disputable gold-standard in microcirculatory explorations.

## Conclusion

Skin temperature around the knee measured with infrared thermography technology is not correlated with mottling score, as the mottling did not directly influence the skin temperature. However, when there is mottling the temperature is lower. This method, compared to standard mottling score is objective and allows data collections. Nevertheless, this method failed to predict mortality in ICU patients. The exact relationship between mottling, skin temperature and ICU-outcomes remain unclear and further investigations are required.

## Supporting information

S1 Table **Skin temperatures in Mottling groups at H0 (A) and H6 (B)**. Temperatures (°C) are expressed in mean ± SD.
(DOC)

## Abbreviations

SOFA: Sequential Organ Failure Assessment
SDF: Sidestream Dark Field
NIRS: Near Infrared Spectroscopy
ICU: Intensive care Unit
SAPS: Simplified Acute Physiology Score
