# Supplementary material for: Mottling score and skin temperature in septic shock: Relation and impact on prognosis in ICU
Source: PLoS One. 2018 Aug 16;13(8):e0202329. doi: 10.1371/journal.pone.0202329 (PMC6095547; doi:10.1371/journal.pone.0202329)
Supplement: S1 Table — Skin temperatures in Mottling groups at H0 (A) and H6 (B). Temperatures (°C) are expressed in mean ± SD. (DOC) [file pone.0202329.s001.doc]

| **Skin temperatures H0** | **Mottling 0** | **Mottling 1** | **Mottling 2** | **Mottling 3** | **Mottling 4** |
| --- | --- | --- | --- | --- | --- |
| **Zone 1** | 31.9 ± 2.1 | 31.1 ± 1.4 | 29.7 ± 1.7 | 31.4 ± 1.9 | 31.4 ± 1.8 |
| **Zone 2** | 32.5 ± 2 ^a^ | 31.4 ± 1.5 | 29.9 ± 1.7 ^a^ | 31.6 ± 1.9 | 31.8 ± 2.1 |
| **Zone 3** | 33 ± 1.9 ^a^ | 32.1 ± 1.6 | 30.5 ± 1.7 ^a^ | 31.9 ± 1.8 | 32 ± 1.5 |
| **Zone 4** | 33.4 ± 2 | 32.8 ± 1.8 | 31.1 ± 1.8 | 32.6 ± 1.8 | 32.5 ± 1.4 |
| **Zone 5** | 34.7 ± 2.1 ^a^ | 33.3 ± 0.9 | 30.8 ± 2.1 ^a^ |  |  |

A.

B.

| **Skin temperatures H6** | **Mottling 0** | **Mottling 1** | **Mottling 2** | **Mottling 3** | **Mottling 4** | **Mottling 5** |
| --- | --- | --- | --- | --- | --- | --- |
| **Zone 1** | 33.3 ± 2.5 ^b^ | 31.2 ± 2.1 | 30.1 ± 1.3 ^b^ | 30.9 ± 1.7 | 30.4 ± 0.4 | 31.8 ± 2.2 |
| **Zone 2** | 33.5 ± 2.5 ^b^ | 31.6 ± 2.1 | 30.5 ± 1.2 ^b^ | 31.3 ± 1.8 | 30.9 ± 0.4 | 32.2 ± 2.2 |
| **Zone 3** | 33.9 ± 2.3 ^b^ | 32.1 ± 1.9 | 30.9 ± 1.1 ^b^ | 31.6 ± 1.9 | 31.5 ± 0.8 | 32.9 ± 2.0 |
| **Zone 4** | 34.7 ± 2.1^b,c^ | 32.6 ± 1.8 | 31.4 ± 0.9^b^ | 31.9 ± 2 ^c^ | 31.6 ± 1.2 | 33.3 ± 1.9 |
| **Zone 5** | 35.1 ± 2.1 ^d^ | 32.2 ± 1.7 ^d^ | 32.6 ± 0.6 |  |  |  |

S1 Table : Skin temperatures in Mottling groups at H0 (A) and H6 (B). Temperatures (°C) are expressed in mean ± SD.

^a^ p≤0.04 between Mottling group 0 and group 2

^b^ p≤0.01 between Mottling group 0 and group 2

^c^ p=0.03 between Mottling group 0 and group 3

^d^ p=0.04 between Mottling group 0 and group 1
